# Cannabis and society: families’ perceptions of cannabis consumption

**DOI:** 10.1192/j.eurpsy.2023.1375

**Published:** 2023-07-19

**Authors:** H. Chebli, F. Laboudi, A. Ouanass

**Affiliations:** University Psychiatric Hospital Arrazi in Sale, Sale, Morocco

## Abstract

**Introduction:**

Cannabis is an illicit substance whose use is very common among the patients we see daily in hospitals. Its use is not simply a “recreational” desire, but above all an attempt at self-medication in order to manage emotions that have become too painful. However, even if cannabis reduces the symptomatology in the moment, it worsens most psychiatric pathologies.

**Objectives:**

The purpose of this study is to assess the experiences of cannabis users’ families.

**Methods:**

The survey was carried out among the families of patients hospitalized at the Arrazi Hospital in Salé and those followed in consultation and who use cannabis. The collection of information is done with the help of an exploitation form.

**Results:**

34.6% of the participating families put bad company as the cause of consumption, followed by family problems, psychiatric problems come in 3rd position with a percentage of 19.2%. Cannabis use is considered as a disease in 52.8% of the families participating in the study. 58.5% of the families distinguish between good and bad cannabis use and define bad use by the use of large quantities of cannabis in 34.2% of the cases. The majority of the participating families (86.8%) saw cannabis as aggravating their loved one’s mental illness.

**Conclusions:**

The understanding of the perceptions of the families towards the use of cannabis by their close relatives as well as the correction of the false perceptions will help to establish better prevention programs and better patient care especially with the family therapy which showed its utility in the management of the patients having disorders related to the use of cannabis.

**Disclosure of Interest:**

None Declared

